# Ego depletion, compensatory health beliefs, and healthy food choice in Chinese university students: a two-wave longitudinal study

**DOI:** 10.3389/fpsyg.2026.1862584

**Published:** 2026-08-05

**Authors:** Jiping He, Xiaoyun Su, Shuyi Jia, Yanming Ren

**Affiliations:** 1 Department of Rehabilitation Medicine, Shanxi University of Medicine, Fenyang, Shanxi, China; 2 Faculty of Health and Wellness, City University of Macau, Macau SAR, China; 3 Department of Nursing, Shanxi University of Medicine, Fenyang, Shanxi, China

**Keywords:** compensatory health beliefs, dietary self-regulation, ego depletion, healthy food choices, longitudinal study, university students

## Abstract

Healthy food choice often requires people to balance immediate reward against longer-term health considerations. The present study examined whether ego depletion and compensatory health beliefs (CHB) were associated with healthy food choice in a two-wave sample of Chinese university students. A total of 394 participants completed measures of ego depletion and CHB and a six-trial binary food-choice task at baseline and again 6 months later. Healthy choices were analyzed both at the trial level using a generalized linear mixed-effects model and at the total-score level using a linear mixed-effects model. At the trial level, higher ego depletion and stronger CHB were both associated with a lower likelihood of choosing the healthier option, whereas the ego depletion × CHB interaction was statistically significant but directionally unexpected and treated as exploratory. A robustness analysis based on the total healthy-choice score replicated the negative main effects, whereas the interaction was only marginally significant. In the longitudinal model, baseline healthy choice was the strongest predictor of follow-up behavior, while ego depletion did not independently predict follow-up choice and CHB showed only a trend-level association. Changes in ego depletion covaried with changes in healthy choice, although the observational change-score analysis does not establish causal direction. Overall, the findings point to substantial stability in dietary decision-making and suggest that ego depletion and CHB have modest, primarily concurrent associations with healthy food choice.

## 1 Introduction

Healthy food choice is often treated as a matter of knowledge or intention, yet actual dietary decisions are commonly made under conflict. In everyday settings, people frequently choose between options that differ in immediate tastiness and longer-term health value, and successful dietary self-control depends in part on how strongly health information is incorporated into the decision process (Hare et al., 2009; Sullivan et al., 2015). This tension is especially relevant in young adults, whose eating patterns are often shaped by convenience, social context, and fluctuating self-control demands (Adriaanse et al., 2014; Boswell et al., 2018).

One factor that may help explain these choices is ego depletion, or more cautiously in the present context, self-regulatory fatigue. The classic strength model proposed that acts of self-control draw on a limited resource and that later regulation can therefore become more difficult after prior effort (Baumeister et al., 2007). Although strong resource-based accounts of ego depletion have been debated, the broader idea that subjective self-regulatory fatigue can undermine health behavior remains influential, especially in domains such as eating where self-control demands are frequent and emotionally salient (Dang, 2018; Hagger et al., 2010). In dietary settings, a depleted or fatigued regulatory state may make it harder for people to maintain the priority of health goals at the point of choice (Hare et al., 2009; Lim et al., 2018).

A second relevant process is compensatory health beliefs (CHB), defined as beliefs that the negative effects of an unhealthy behavior can later be offset by a healthy one (Knäuper et al., 2004). In the eating domain, CHB are psychologically attractive because they allow people to preserve both immediate gratification and a positive health self-image. Prior research suggests that CHB can hinder eating behavior change and are particularly relevant in contexts involving unhealthy snacks and indulgent foods (Amrein et al., 2017; Amrein et al., 2021). Rather than reflecting simple ignorance, such beliefs may serve as a form of self-licensing that reduces the immediate psychological cost of choosing a less healthy option (Knäuper et al., 2004; Zhao et al., 2021).

These two processes may also work together. When people feel mentally tired or behaviorally strained, they may not only have more difficulty acting in line with health goals, but may also be more inclined to rationalize unhealthy choices. Under those conditions, CHB may make it easier to justify indulgence by framing it as temporary or repairable. Even so, the empirical literature has more often examined ego depletion and CHB separately than together, and relatively few studies have linked both constructs to repeated behavioral food-choice data collected over time (Amrein et al., 2017; Bailey and Lim, 2024).

The present study addressed this gap using a two-wave design and a six-trial food-choice task in a sample of Chinese university students. Healthy choice was modeled both at the trial level and at the total-score level. We expected that higher ego depletion and stronger CHB would each be associated with fewer healthy choices. We also examined their interaction, while treating it as theoretically plausible but empirically secondary. Finally, because concurrent and prospective effects do not always align in self-regulation research, we tested not only baseline-to-follow-up prediction but also whether changes in ego depletion and CHB tracked changes in healthy food choice over time.

## 2 Materials and methods

### 2.1 Participants and procedure

**Figure 1:**
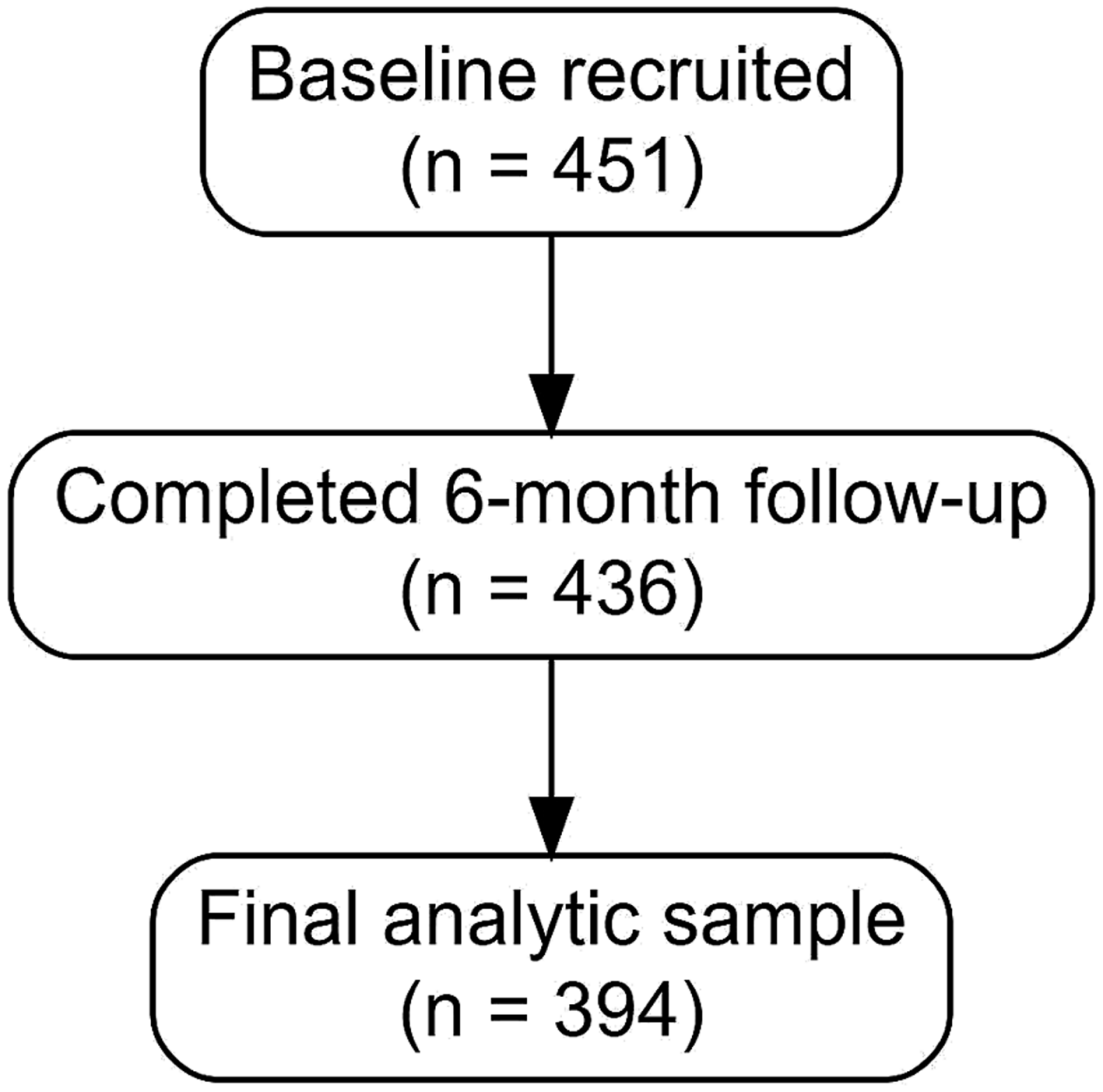
Participant flow chart.

At baseline (Time 1, T1), we recruited a total of 451 undergraduate students from a university in Anhui Province, China. Follow-up data collection was conducted at the class level approximately 6 months later, and 436 responses were obtained at T2. Because some baseline participants did not complete the follow-up assessment and some follow-up responses could not be validly matched to baseline records, only participants with valid matched responses at both T1 and T2 were eligible for the longitudinal analyses. Before formal model estimation, the two-wave dataset was screened using data-quality criteria that required completion of the focal measures and correct responses to attention-check items. The final analytic sample consisted of 394 participants, with an effective overall retention rate of 87.4% (see Figure 1).

The final sample had a mean age of 21.37 years (SD = 0.93, range = 19.00–27.00) and a mean body mass index (BMI) of 23.07 (SD = 4.46, range = 14.87–41.04). The sample included 189 male participants and 205 female participants. Place of residence was also recorded at baseline. Written informed consent was obtained from all participants prior to data collection. The study protocol was approved by the Institutional Review Board of the authors’ affiliated institution.

At both T1 and T2, participants completed self-report measures of ego depletion and compensatory health beliefs (CHB), followed by a six-trial food-choice task. In each trial, participants were presented with one relatively healthier food option and one relatively less healthy food option and were asked to select the option they would be more likely to choose in their future diet. The six food pairs used in the formal study were selected in a preliminary material-screening study.

### 2.2 Measures

#### 2.2.1 Ego depletion

Ego depletion (i.e., self-regulatory fatigue) was assessed at both time points using the Chinese version of the 16-item Self-Regulatory Fatigue Scale (SRF-S; Nes et al., 2013; Wang et al., 2015). Participants rated each item on a 5-point Likert scale, with anchors ranging from 1 = strongly disagree to 5 = strongly agree. Prior to scoring, 6 positively phrased items were reverse-coded to align with the scale’s scoring logic (higher scores reflect greater self-regulatory fatigue). A total self-regulatory fatigue score was calculated for each wave by summing all item scores, with a theoretical score range of 16 to 80. The scale demonstrated good internal consistency reliability at both assessments (T1: Cronbach’s *α* = 0.854; T2: Cronbach’s *α* = 0.864).

#### 2.2.2 Compensatory health beliefs

Compensatory health beliefs, defined as the beliefs that the negative effects of unhealthy behaviors can be neutralized by engaging in healthy behaviors, were assessed at both time points using the 6-item short form of the Compensatory Health Beliefs Scale (CHB Scale; Knäuper et al., 2004). This short-form scale has been widely validated and applied in longitudinal health psychology research, with well-documented reliability and validity in Chinese samples (Zhang et al., 2022). Participants rated each item on a 5-point Likert scale, with anchors ranging from 1 = strongly disagree to 5 = strongly agree. Consistent with established scoring protocols for this scale, a mean score was calculated by averaging responses across all six items for each wave, with higher scores reflecting stronger endorsement of compensatory health beliefs. The scale demonstrated good internal consistency reliability at both assessments (T1: Cronbach’s *α* = 0.834; T2: Cronbach’s *α* = 0.865).

#### 2.2.3 Healthy food-choice task

Healthy dietary decision-making was assessed using a 6-trial forced-choice paradigm, which was developed and validated for the Chinese dietary context via pilot work conducted as part of the present study. In the pilot phase, nine pairs of food images matched by food category were initially selected from a standardized Chinese food picture library (Cai et al., 2023). We recruited 30 participants to rate each food on perceived palatability and healthiness using a 9-point Likert scale. Six pairs of food stimuli were retained for the formal task, with paired-samples t-tests confirming significant differences in both perceived healthiness and palatability between the healthier and less healthy options within each pair (all *ps* < 0.05). Each trial of the formal task presented one pair of images, consisting of one nutritionally healthier option and one energy-dense, less healthy option from the same food category.

During the task, participants were instructed to select which option they would be more likely to consume in their daily diet for each trial. At the trial level, the choice of the healthier option was coded as 1, and the choice of the less healthy option was coded as 0. A total healthy dietary decision score was calculated by summing the coded values across all six trials, resulting in a possible score range of 0 to 6, with higher scores indicating a greater propensity to make healthy food choices. Full details of the pilot test results and food stimuli are provided in the Supplementary materials.

#### 2.2.4 Covariates

Baseline demographic characteristics including age, gender, residential area, height, and weight were collected via self-report at the baseline assessment. Body mass index (BMI) was calculated as weight in kilograms divided by the square of height in meters (kg/m^2^). Consistent with standard practice in dietary behavior research with a 6-month follow-up period, BMI was treated as a time-invariant covariate and entered into all multivariable models. Height and weight were only assessed at baseline, as the primary outcomes of this study focused on dietary decision-making and compensatory health beliefs (rather than weight change), and within-person fluctuations in BMI among adults over the 6-month study period are negligible and do not affect the control of baseline individual differences in weight status.

### 2.3 Statistical analysis

All analyses were conducted in R [4.3.3]. The final analytic sample included 394 participants with complete data on the variables entered into the primary models. Descriptive statistics and internal consistency coefficients were first computed for all focal variables. Paired-samples *t*-tests were then used to examine changes from T1 to T2 in ego depletion, CHB, and healthy food-choice scores. Pearson correlations were used to examine bivariate associations among the focal variables.

To test the primary hypotheses at the behavioral level, a generalized linear mixed-effects model (GLMM) with a logit link was fitted to the trial-level food-choice data. Healthy choice (1 = healthier option chosen, 0 = less healthy option chosen) was treated as the dependent variable. Time, standardized ego depletion, standardized CHB, and their interaction were entered as fixed effects. Participant and task were modeled as random intercepts. Age and BMI were standardized prior to model estimation, and sex and residence were entered as covariates.

As a robustness check, a linear mixed-effects model (LMM) was estimated using the total healthy decision score (0–6) across the two waves. This model included the same focal predictors and covariates, with participant modeled as a random intercept. Because the healthy food-choice total score was bounded from 0 to 6, this total-score LMM was treated as a secondary robustness analysis. Primary inference was based on the trial-level GLMM, which modeled each binary food-choice decision directly; residual diagnostics for the LMM did not indicate severe violations of model assumptions.

To examine longitudinal prediction, a regression model was fitted with T2 healthy decision score as the outcome, while controlling for T1 healthy decision score. Baseline ego depletion, baseline CHB, and their interaction were included as predictors. Finally, a change-score model was estimated to examine whether within-person changes in ego depletion and CHB were associated with changes in healthy food-choice behavior over the 6-month interval. Two-tailed *p* values below 0.05 were considered statistically significant. The trial-level GLMM was specified as the primary model for behavioral inference. The total-score LMM, prospective model, change-score model, and interaction findings were treated as complementary or exploratory analyses. No formal familywise error-rate adjustment was applied across these complementary tests, and secondary findings were interpreted in light of effect sizes, confidence intervals, and consistency across models.

## 3 Results

### 3.1 Preliminary analyses

Table 1 summarizes the demographic characteristics, descriptive statistics, and internal consistency of all key measures. Both the self-regulatory fatigue (ego depletion) scale and the compensatory health beliefs (CHB) scale demonstrated good internal consistency across the two waves of assessment (ego depletion: T1 *α* = 0.854, T2 α = 0.864; CHB: T1 α = 0.834, T2 α = 0.865). On average, participants reported lower ego depletion at the 6-month follow-up (T2: *M* = 37.82, *SD* = 9.48) compared to baseline (T1: *M* = 39.04, *SD* = 9.37), while CHB scores remained stable across waves (T1: *M* = 2.69, *SD* = 0.88; T2: *M* = 2.70, *SD* = 0.88). Healthy dietary decision scores increased slightly but significantly from baseline (*M* = 4.01, *SD* = 1.51) to follow-up (*M* = 4.14, *SD* = 1.51).

**Table 1:** Demographic characteristics, descriptive statistics, and internal consistency coefficients of key variables.

Variable	*n*	*M* (*SD*)	Range	Cronbach’s *α*
Age (years)	394	21.37 (0.93)	19–27	—
BMI	394	23.07 (4.46)	14.87–41.04	—
Ego depletion T1	394	39.04 (9.37)	16–62	0.854
Ego depletion T2	394	37.82 (9.48)	16–62	0.864
CHB T1	394	2.69 (0.88)	1–5	0.834
CHB T2	394	2.70 (0.88)	1–5	0.865
Healthy decisions T1 (0–6)	394	4.01 (1.51)	0–6	—
Healthy decisions T2 (0–6)	394	4.14 (1.51)	0–6	—

CHB, compensatory health beliefs. Healthy decisions represent the number of healthy choices out of six dietary decision trials.

We conducted paired-samples t-tests to examine within-person changes over time. Ego depletion decreased between T1 and T2, *t*(393) = 3.47, *p* < 0.001, Cohen’s d = 0.13, whereas the mean level of CHB remained unchanged, *t*(393) = −0.08, *p* = 0.936, Cohen’s d = −0.01. Healthy dietary decision scores increased over the 6-month interval, *t*(393) = −2.07, *p* = 0.039, Cohen’s d = −0.09. These temporal changes were statistically detectable but small in magnitude.

Bivariate Pearson correlations (full results in Table 2) aligned with our hypothesized associations: healthy dietary decision scores were significantly negatively correlated with ego depletion and CHB at both baseline and follow-up. Ego depletion also showed a consistent positive correlation with CHB across both waves.

**Table 2:** Pearson correlations among the focal variables.

Variable	1	2	3	4	5	6	7	8
1. Age	—							
2. BMI	0.05	—						
3. Ego depletion T1	−0.08	−0.01	—					
4. Ego depletion T2	−0.08	0.01	0.73^***^	—				
5. CHB T1	−0.03	−0.08	0.23^***^	0.19^***^	—			
6. CHB T2	−0.03	−0.11^*^	0.20^***^	0.25^***^	0.55^***^	—		
7. Healthy decisions T1	0.10	0.04	−0.19^***^	−0.14^**^	−0.19^***^	−0.13^**^	—	
8. Healthy decisions T2	0.12^*^	0.07	−0.15^**^	−0.22^***^	−0.20^***^	−0.20^***^	0.65^***^	—

^*^p < 0.05, ^**^p < 0.01, ^***^p < 0.001.

### 3.2 Trial-level mixed-effects model of healthy food choice

The primary generalized linear mixed-effects model (GLMM) predicting trial-level healthy food choice is presented in Table 3. As hypothesized, higher ego depletion was associated with a significantly lower likelihood of choosing the healthier food option, *b* = −0.286, *SE* = 0.058, *z* = −4.92, *p* < 0.001, *OR* = 0.751, 95% CI [0.670, 0.842]. Higher CHB was also associated with a significantly lower likelihood of healthy food choice, *b* = −0.180, *SE* = 0.054, *z* = −3.34, *p* < 0.001, *OR* = 0.836, 95% CI [0.752, 0.928].

**Table 3:** Trial-level GLMM predicting binary healthy food choice (*N* = 394 participants, 4,728 total trials).

Predictor	*b*	*SE*	*z*	*p*	Odds ratio	95% CI for *OR*
Intercept	0.855	0.227	3.77	<0.001	2.352	[1.508, 3.668]
Time (T2 vs. T1)	0.092	0.070	1.33	0.185	1.097	[0.957, 1.257]
Ego depletion (standardized)	−0.286	0.058	−4.92	<0.001	0.751	[0.670, 0.842]
CHB (standardized)	−0.180	0.054	−3.34	<0.001	0.836	[0.752, 0.928]
Ego depletion × CHB	0.103	0.049	2.09	0.036	1.109	[1.007, 1.221]
Age (standardized)	0.151	0.065	2.33	0.020	1.163	[1.024, 1.321]
Sex (female vs. male)	0.189	0.142	1.33	0.182	1.208	[0.916, 1.592]
Residence (urban vs. rural)	−0.100	0.128	−0.78	0.438	0.905	[0.704, 1.164]
BMI (standardized)	0.099	0.068	1.45	0.147	1.104	[0.966, 1.263]

Random intercepts for participants and tasks were included in the model.

The main effect of time was not statistically significant, *b* = 0.092, *SE* = 0.070, *z* = 1.33, *p* = 0.185, indicating no overall change in healthy food-choice probability between baseline and follow-up after accounting for predictors and covariates. The ego depletion × CHB interaction was statistically significant at the trial level, *b* = 0.103, *SE* = 0.049, *z* = 2.09, *p* = 0.036, OR = 1.109, 95% CI [1.007, 1.221]. The positive interaction coefficient indicated that the negative association between ego depletion and healthy choice was steeper at lower, rather than higher, levels of CHB. Because this direction was not consistent with the hypothesized amplification pattern, and because the interaction was not replicated in the total-score robustness model, this pattern was treated as exploratory. A descriptive plot of the interaction is provided in Supplementary Figure S1.

### 3.3 Total-score mixed-effects model

To evaluate the robustness of the trial-level findings, a linear mixed-effects model was fitted to the total healthy decision score. As shown in Table 4, higher ego depletion significantly predicted lower healthy decision scores, *b* = −0.279, *SE* = 0.056, *t* = −4.97, *p* < 0.001, 95% CI [−0.389, −0.169]. Higher CHB also significantly predicted lower healthy decision scores, *b* = −0.160, *SE* = 0.051, *t* = −3.14, *p* = 0.002, 95% CI [−0.260, −0.060].

**Table 4:** Total-score linear mixed-effects model predicting healthy decision score.

Predictor	*b*	*SE*	*t*	*p*	95% CI
Intercept	3.944	0.129	30.62	<0.001	[3.691, 4.197]
Time (T2 vs. T1)	0.095	0.063	1.51	0.131	[−0.028, 0.218]
Ego depletion (standardized)	−0.279	0.056	−4.97	<0.001	[−0.389, −0.169]
CHB (standardized)	−0.160	0.051	−3.14	0.002	[−0.260, −0.060]
Age (standardized)	0.150	0.068	2.22	0.027	[0.017, 0.283]
Sex (female vs. male)	0.226	0.149	1.52	0.129	[−0.066, 0.519]
Residence (urban vs. rural)	−0.102	0.134	−0.76	0.447	[−0.366, 0.162]
BMI (standardized)	0.109	0.072	1.50	0.134	[−0.034, 0.251]
Ego depletion × CHB	0.081	0.045	1.80	0.072	[−0.007, 0.169]

The main effect of time was not significant in this model, *b* = 0.095, *SE* = 0.063, *t* = 1.51, *p* = 0.131. The ego depletion × CHB interaction was not statistically significant, *b* = 0.081, *SE* = 0.045, *t* = 1.80, *p* = 0.072, 95% CI [−0.007, 0.169]. Taken together, these findings suggest that the negative main effects of ego depletion and CHB on healthy food choice were robust across analytic approaches, whereas the interaction effect received only partial support.

### 3.4 Longitudinal prediction of healthy food choice

A longitudinal regression model was then estimated to examine whether baseline ego depletion and CHB predicted T2 healthy food-choice scores after controlling for T1 healthy food-choice scores. As shown in Table 5, baseline healthy food-choice behavior was the largest and most precise predictor of T2 behavior, *b* = 0.628, *SE* = 0.040, *t* = 15.73, *p* < 0.001, 95% CI [0.550, 0.707], indicating substantial temporal stability in dietary decision-making.

**Table 5:** Longitudinal regression model predicting T2 healthy food-choice scores.

Predictor	*b*	*SE*	*t*	*p*	95% CI
Intercept	1.731	0.188	9.19	<0.001	[1.361, 2.102]
Healthy decisions T1	0.628	0.040	15.73	<0.001	[0.550, 0.707]
Ego depletion T1 (standardized)	−0.016	0.061	−0.26	0.794	[−0.135, 0.104]
CHB T1 (standardized)	−0.102	0.062	−1.66	0.098	[−0.223, 0.019]
Age (standardized)	0.076	0.059	1.29	0.199	[−0.040, 0.193]
Sex (female vs. male)	−0.026	0.131	−0.20	0.842	[−0.285, 0.232]
Residence (urban vs. rural)	−0.172	0.117	−1.48	0.141	[−0.402, 0.057]
BMI (standardized)	0.056	0.063	0.89	0.372	[−0.068, 0.181]
Ego depletion T1 × CHB T1	−0.002	0.058	−0.03	0.975	[−0.116, 0.112]

After adjustment for baseline healthy food-choice scores and covariates, baseline ego depletion did not significantly predict T2 healthy food-choice scores, *b* = −0.016, *SE* = 0.061, *t* = −0.26, *p* = 0.794, 95% CI [−0.135, 0.104]. Baseline CHB also did not significantly predict T2 healthy food-choice scores, although the coefficient was negative, *b* = −0.102, *SE* = 0.062, *t* = −1.66, *p* = 0.098, 95% CI [−0.223, 0.019]. The baseline ego depletion × CHB interaction was not significant, *b* = −0.002, *SE* = 0.058, *t* = −0.03, *p* = 0.975, 95% CI [−0.116, 0.112]. Thus, the principal longitudinal finding was the temporal stability of healthy food choice. Once prior behavior was accounted for, the incremental prospective contributions of baseline ego depletion and CHB were limited and statistically uncertain.

### 3.5 Change-score analysis

Finally, a change-score model was estimated to examine whether within-person changes in ego depletion and CHB were associated with changes in healthy food-choice behavior over the 6-month interval. As shown in Table 6, a greater increase in ego depletion was associated with a greater decline in healthy food-choice behavior, *b* = −0.230, *SE* = 0.065, *t* = −3.53, *p* < 0.001, 95% CI [−0.358, −0.102]. In contrast, change in CHB was not significantly associated with change in healthy food-choice behavior, *b* = −0.069, *SE* = 0.064, *t* = −1.09, *p* = 0.276, 95% CI [−0.194, 0.056]. The interaction between changes in ego depletion and CHB was also not significant, *b* = 0.020, *SE* = 0.052, *t* = 0.39, *p* = 0.697, 95% CI [−0.082, 0.123].

**Table 6:** Change-score model predicting change in healthy food-choice behavior across 6 months.

Predictor	*b*	*SE*	*t*	*p*	95% CI
Intercept	0.275	0.117	2.34	0.02	[0.044, 0.505]
ΔEgo depletion (standardized)	−0.230	0.065	−3.53	<0.001	[−0.358, −0.102]
ΔCHB (standardized)	−0.069	0.064	−1.09	0.276	[−0.194, 0.056]
Age (standardized)	0.023	0.065	0.36	0.721	[−0.104, 0.150]
Sex (female vs. male)	−0.111	0.140	−0.79	0.430	[−0.386, 0.165]
Residence (urban vs. rural)	−0.155	0.127	−1.23	0.220	[−0.405, 0.094]
BMI (standardized)	0.032	0.069	0.46	0.645	[−0.104, 0.168]
ΔEgo depletion × ΔCHB	0.020	0.052	0.39	0.697	[−0.082, 0.123]

Positive change scores indicate increases from T1 to T2. Negative coefficients indicate that increases in the predictor were associated with decreases in healthy food-choice behavior.

**Figure 2:**
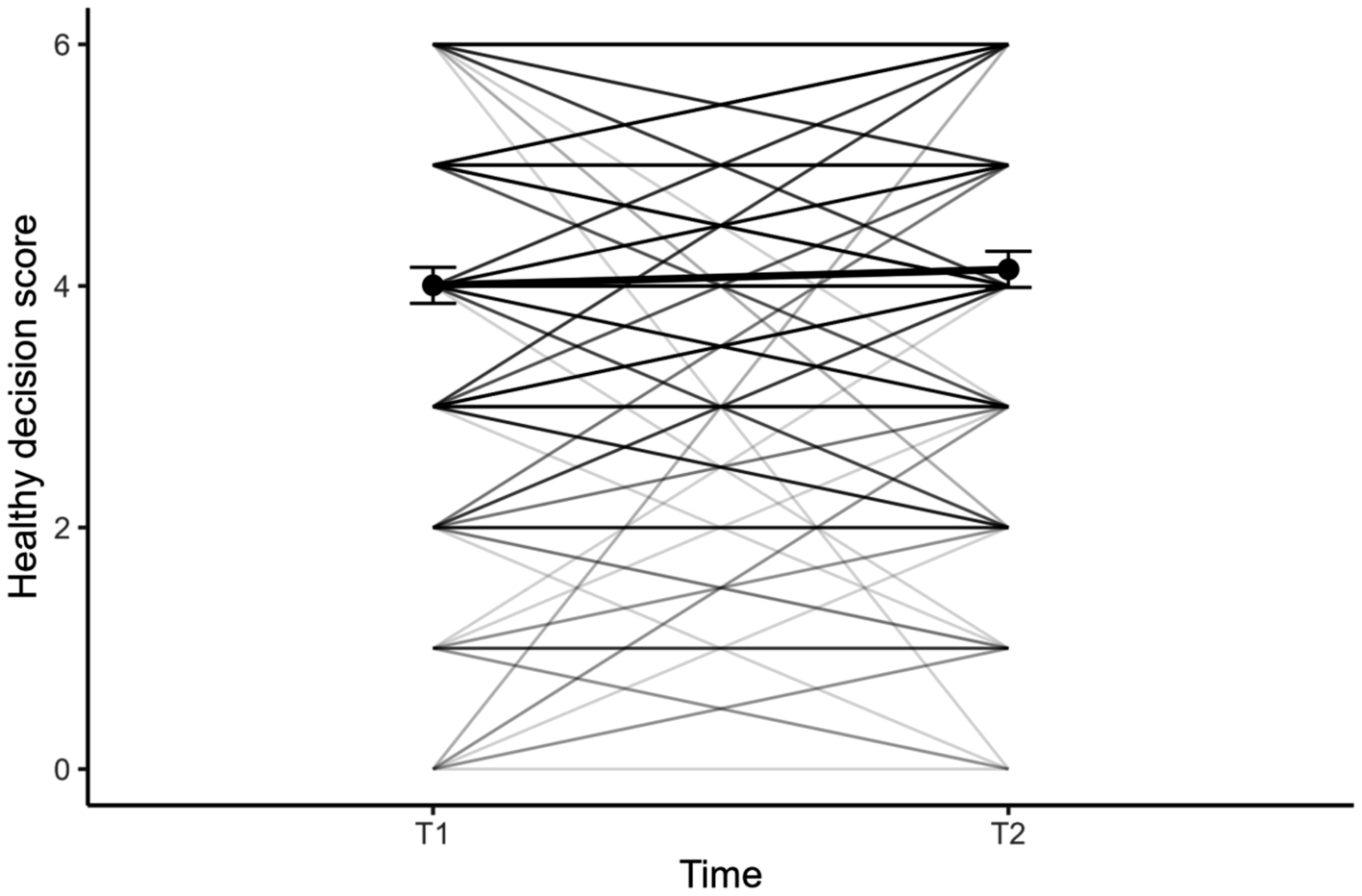
Mean healthy decision scores at T1 and T2 with 95% confidence intervals. Error bars represent 95% confidence intervals.

Overall, the change-score analysis identified covariation between changes in ego depletion and changes in healthy food-choice behavior. This association should not be interpreted causally: the two-wave observational design cannot determine whether changes in self-regulatory fatigue preceded dietary change, whether dietary change influenced perceived fatigue, or whether both reflected unmeasured time-varying factors (see Figure 2).

## 4 Discussion

The present study examined whether ego depletion and compensatory health beliefs were associated with healthy food choice in a two-wave sample of university students. The most prominent longitudinal finding was the substantial stability of healthy food choice from baseline to follow-up. Against this stable behavioral background, ego depletion and CHB showed modest concurrent associations with less healthy choice, whereas their incremental prospective contributions were limited after prior behavior was taken into account. The ego depletion × CHB interaction appeared at the trial level but was directionally unexpected, weakened in the total-score model, and did not extend to the longitudinal models. The discussion therefore focuses on behavioral stability, the replicated concurrent main effects, and the more tentative status of the interaction and change-score findings.

The negative association between ego depletion and healthy food choice is theoretically plausible and consistent with the broader literature on dietary self-control. Healthy eating often requires the person to weigh health information against immediate reward, and prior work has shown that successful self-control in food choice depends on the extent to which health attributes remain behaviorally salient during decision making (Hare et al., 2009; Sullivan et al., 2015). In that sense, the present findings suggest that self-regulatory fatigue may not simply reflect a general sense of tiredness; it may index moments in which people are less able to keep long-term health goals active when facing tempting options (Baumeister et al., 2007; Lim et al., 2018).

The CHB findings point to a related but distinct process. Stronger compensatory beliefs were associated with less healthy choice across the concurrent models, suggesting that these beliefs may function as a cognitive permission structure for unhealthy decisions. This interpretation is consistent with the original CHB framework and with later work showing that such beliefs can interfere with eating behavior change and everyday dietary restraint (Knäuper et al., 2004; Amrein et al., 2017; Amrein et al., 2021). In practical terms, CHB may reduce guilt in the moment of choice by allowing the person to think that an unhealthy act can later be offset or repaired.

The ego depletion × CHB interaction warrants particular caution. The plotted pattern indicated that the negative association between ego depletion and healthy choice was more pronounced when CHB was low, not high. This direction is counter to the hypothesis that compensatory beliefs would amplify the behavioral consequences of depletion. One possible explanation is that higher CHB was already associated with a lower probability of healthy choice, leaving less room for ego depletion to further differentiate responses; however, this *post hoc* explanation was not directly tested. Given the marginal replication in the total-score model, the lack of evidence in the longitudinal models, and the absence of familywise error-rate adjustment across complementary tests, the interaction should be interpreted as an exploratory pattern rather than evidence for the proposed synergistic mechanism.

The longitudinal findings place these psychological associations in perspective. Baseline healthy choice strongly predicted later healthy choice, whereas baseline ego depletion did not add prospective explanatory value and CHB contributed only a statistically uncertain increment. Such stability may reflect consolidated routines, repeated exposure to similar food environments, relatively stable preferences, or dispositional tendencies. Habit accounts propose that repeated behavior in stable contexts becomes increasingly automatic and cue dependent (Verplanken and Orbell, 2003; Wood and Neal, 2007), and meta-analytic evidence indicates that habit contributes to the continuity of health behavior alongside behavioral intentions (Hagger et al., 2023). Research on healthy eating likewise shows that intentional and motivational processes contribute to subsequent dietary behavior (Girelli et al., 2016). Because the present study did not directly measure habit strength, intentions, or environmental stability, these mechanisms cannot be distinguished. Nevertheless, the results indicate that prior dietary behavior accounted for substantially more of later choice than the baseline psychological vulnerability measures examined here.

The different mean-level trajectories of the two predictors also have theoretical implications. Ego depletion decreased slightly across 6 months, whereas CHB remained stable. This pattern is consistent with greater temporal sensitivity in self-regulatory fatigue, which has been conceptualized as a temporary reduction in regulatory capacity or a fatigue state following self-regulatory demands (Baumeister et al., 1998; Evans et al., 2016). By contrast, compensatory health beliefs refer to health-related beliefs used to reconcile conflict between immediate desires and longer-term health goals (Knäuper et al., 2004; Rabiau et al., 2006), and their stable mean may reflect a comparatively stable belief structure. However, two waves of mean-level data cannot establish a strict state–trait distinction. In the change-score model, increases in ego depletion were associated with declines in healthy choice, whereas changes in CHB were not. This result represents within-person covariation rather than evidence that self-regulatory fatigue caused dietary deterioration. Reverse or reciprocal pathways remain plausible: less healthy eating could increase the subjective burden of self-regulation, and unmeasured changes in stress or daily context could influence both variables. Stronger causal and temporal tests would require additional waves or designs that manipulate self-regulatory demands.

The practical implications should be considered in light of the modest effect sizes. The mean changes across waves were small (|d| = 0.01–0.13), and a 1-SD increase in ego depletion or CHB corresponded to odds ratios of 0.751 and 0.836, respectively, in a brief experimental choice task. These estimates may be relevant at the population level or across repeated daily decisions, but they do not imply large changes in an individual’s diet. Interventions that simplify healthy choices, reduce decision burden, or challenge compensatory beliefs may therefore be worth testing, but the present findings alone do not establish substantial applied benefit (Boswell et al., 2018; Rosenblatt et al., 2018).

This study has several limitations. The sample was restricted to university students with a relatively narrow age range, which limits generalizability. The analytic sample included only participants with valid matched responses at both waves who met the data-quality criteria; findings may therefore not generalize to participants who did not complete follow-up or whose responses could not be matched. The two-wave design cannot establish causal direction or characterize longer-term developmental trajectories, and the change-score analysis should be understood as associational rather than causal (Tennant et al., 2022). The food-choice task captured behavioral preference but could not reproduce the complexity of everyday eating environments, and the psychological predictors were self-reported. Finally, no formal familywise error-rate adjustment was applied. Although the trial-level GLMM was specified as the primary model and the remaining analyses were interpreted as complementary or exploratory evidence, false-positive findings remain possible, particularly for the interaction. These limitations reinforce the need to interpret the psychological effects as modest and provisional relative to the strong temporal stability of healthy food choice.

## 5 Conclusion

Healthy food choice showed substantial temporal stability across 6 months. Higher ego depletion and stronger compensatory health beliefs had modest concurrent associations with less healthy choice, but their incremental prospective contributions were limited after prior behavior was considered. Changes in ego depletion covaried with changes in healthy choice, although causal direction cannot be inferred, and the ego depletion × CHB interaction was exploratory, directionally unexpected, and not robust across models. The findings therefore support a cautious account in which stable behavioral patterns are primary and self-regulatory fatigue and compensatory beliefs may contribute at or near the point of choice.

## Data Availability

The raw data supporting the conclusions of this article will be made available by the authors, without undue reservation.
