# Supplementary material for: Ego depletion, compensatory health beliefs, and healthy food choice in Chinese university students: a two-wave longitudinal study
Source: Front Psychol. 2026 Aug 5;17:1862584. doi: 10.3389/fpsyg.2026.1862584 (PMC13485565; doi:10.3389/fpsyg.2026.1862584)
Supplement: Supplementary file 1 [file Supplementary_file_1.DOCX]

**Supplementary Figure S1**. Exploratory interaction of ego depletion and CHB on healthy food choice.

**Note.** Predicted probabilities of choosing the healthy option as a function of standardized ego depletion at low (−1 SD), mean, and high (+1 SD) levels of compensatory health beliefs (CHB). Shaded areas indicate 95% confidence intervals. This figure provides a descriptive illustration of the exploratory interaction and should not be interpreted as confirmatory evidence of moderation.
